# Multiple radiations of spiny mice (Rodentia: *Acomys*) in dry open habitats of Afro-Arabia: evidence from a multi-locus phylogeny

**DOI:** 10.1186/s12862-019-1380-9

**Published:** 2019-03-04

**Authors:** T. Aghová, K. Palupčíková, R. Šumbera, D. Frynta, L. A. Lavrenchenko, Y. Meheretu, J. Sádlová, J. Votýpka, J. S. Mbau, D. Modrý, J. Bryja

**Affiliations:** 1 Institute of Vertebrate Biology of the Czech Academy of Sciences, 603 65 Brno, Czech Republic; 2 Department of Zoology, National Museum, 115 79 Prague, Czech Republic; 3 Department of Zoology, Faculty of Science, Charles University, 128 44 Prague, Czech Republic; 4 Department of Zoology, Faculty of Science, University of South Bohemia, 370 05 České Budějovice, Czech Republic; 5 A. N. Severtsov Institute of Ecology and Evolution RAS, 119071 Moscow, Russia; 6 Department of Biology and Institute of Mountain Research and Development, Mekelle University, P.O. Box 3102, Mekelle, Tigray Ethiopia; 7 Department of Parasitology, Faculty of Science, Charles University, 128 44 Prague, Czech Republic; 8 Institute of Parasitology, Biology Centre of the Czech Academy of Sciences, 370 05 České Budějovice, Czech Republic; 9 Department of Land Resource Management and Agricultural Technology, College of Agriculture and Veterinary Sciences, University of Nairobi, Nairobi, Kenya; 10 Department of Pathology and Parasitology, Faculty of Veterinary Medicine, University of Veterinary and Pharmaceutical Sciences, 612 42 Brno, Czech Republic; 11 Department of Botany and Zoology, Faculty of Science, Masaryk University, 602 00 Brno, Czech Republic

**Keywords:** *Acomys*, Savanna, Biogeography, Africa, Arabia, Sahara, Somali-Masai, Zambezian savanna, Plio-Pleistocene

## Abstract

**Background:**

Spiny mice of the genus *Acomys* are distributed mainly in dry open habitats in Africa and the Middle East, and they are widely used as model taxa for various biological disciplines (e.g. ecology, physiology and evolutionary biology). Despite their importance, large distribution and abundance in local communities, the phylogeny and the species limits in the genus are poorly resolved, and this is especially true for sub-Saharan taxa. The main aims of this study are (1) to reconstruct phylogenetic relationships of *Acomys* based on the largest available multilocus dataset (700 genotyped individuals from 282 localities), (2) to identify the main biogeographical divides in the distribution of *Acomys* diversity in dry open habitats in Afro-Arabia, (3) to reconstruct the historical biogeography of the genus, and finally (4) to estimate the species richness of the genus by application of the phylogenetic species concept.

**Results:**

The multilocus phylogeny based on four genetic markers shows presence of five major groups of *Acomys* called here *subspinosus, spinosissimus, russatus, wilsoni* and *cahirinus* groups. Three of these major groups (*spinosissimus, wilsoni* and *cahirinus*) are further sub-structured to phylogenetic lineages with predominantly parapatric distributions. Combination of alternative species delimitation methods suggests the existence of 26 molecular operational taxonomic units (MOTUs), potentially corresponding to separate species. The highest genetic diversity was found in Eastern Africa. The origin of the genus *Acomys* is dated to late Miocene (*ca.* 8.7 Ma), when the first split occurred between spiny mice of eastern (Somali-Masai) and south-eastern (Zambezian) savannas. Further diversification, mostly in Plio-Pleistocene, and the current distribution of *Acomys* were influenced by the interplay of global climatic factors (e.g.*,* Messinian salinity crisis, intensification of Northern Hemisphere glaciation) with local geomorphology (mountain chains, aridity belts, water bodies). Combination of divergence dating, species distribution modelling and historical biogeography analysis suggests repeated “out-of-East-Africa” dispersal events into western Africa, the Mediterranean region and Arabia.

**Conclusions:**

The genus *Acomys* is very suitable model for historical phylogeographic and biogeographic reconstructions of dry non-forested environments in Afro-Arabia. We provide the most thorough phylogenetic reconstruction of the genus and identify major factors that influenced its evolutionary history since the late Miocene. We also highlight the urgent need of integrative taxonomic revision of east African taxa.

**Electronic supplementary material:**

The online version of this article (10.1186/s12862-019-1380-9) contains supplementary material, which is available to authorized users.

## Background

The Old-World savanna biome spans the tropical grasslands, scrublands and wooded savannas of sub-Saharan Africa [1]. These open non-forested habitats represent the most widespread terrestrial environment in Africa [2] and they harbour one of the most abundant and diverse mammalian communities on Earth [3]. In Africa, four major biogeographic regions can be distinguished, which are defined by the geographical distribution of vascular plants and terrestrial vertebrates, where savanna-like ecosystems predominate (Zambezian, Somali, Sudanian and South African [3]).

Numerous geological and climatic events have affected the biological diversity of contemporary savanna-like ecosystems in Africa. In Eastern Africa, the East African Rift System (EARS) started to develop *ca.* 45–33 Ma [4], which led to a change in the region’s topography and the consequent aridification of East Africa, most intensively since late Miocene [5–8]. These climatic changes are best documented by the shift from C3 (moisture-adapted plants) to C4 (tropical arid-adapted grasses) plants [9–11]. The climatically turbulent Pliocene and especially Pleistocene periods, when arid and humid conditions alternated, resulted in a series of expansions and contractions of climatic zones [12, 13] that influenced the distribution and diversification of biodiversity in this region [14, 15].

Small mammals, especially rodents, are very good model organisms for phylogeographic reconstructions. Most of them are usually habitat specialists, exhibit low dispersal ability and have relatively high substitution rates, at least at mitochondrial DNA (mtDNA). Spiny mice of the genus *Acomys* I. Geoffroy Saint-Hilaire, 1838 inhabit seasonally dry open habitats in large regions of sub-Saharan Africa, the Eastern Mediterranean and the Arabian Peninsula [16]. Because they usually constitute abundant parts of local small mammal communities and their samples are easy to collect, they potentially represent a suitable group for testing hypotheses pertaining to the biogeography of dry open habitats in Africa and Arabia. *Acomys* belongs to a handful of rodent taxa that have been extensively studied for decades, and they have been used in several fields of study (e.g. ecology [17–22], physiology [23–26] and evolutionary biology [27–29]). Nevertheless, the vast majority of these studies was performed on taxa from Israel and neighbouring areas of the Middle East, representing only small fragment of the phylogenetic diversity of the genus [30–33].

The genus *Acomys* was described as a separate taxon at the beginning of the nineteenth century, but there is still no synthesis of diversity across the genus, even though species names and descriptions abound [16, 34–38] (see Additional file 1). There were repeated attempts for systematic classification of *Acomys* using morphological characters [34, 39] and chromosomes [38, 40] (see Additional file 1). However, many currently recognized species cannot be easily distinguished using morphological characters due to significant intraspecific variability and generalized morphology. Available molecular studies [30–33, 41–46], are all based on limited taxon sampling and/or geographic coverage. Furthermore, earlier studies largely relied only on sequences of mitochondrial genes, which can be misleading in species delimitations (e.g.*,* [47, 48]). To conclude, the estimation of the total number and delimitation of extant *Acomys* species and their biogeographical history would benefit from a more extensive study based on multiple molecular markers.

### Aims

In this study, we focus on phylogeography and biogeography of the genus *Acomys* by phylogenetic analysis of the largest available dataset and substantially improved geographic and taxon sampling. The aims of this study are (1) to reconstruct the phylogeny of the genus using multilocus dataset; (2) to identify the main biogeographical divides in the distribution of *Acomys* diversity in seasonally dry open habitats in Afro-Arabia; (3) to test proposed hypotheses of historical biogeography of the genus *Acomys* (i.e. to disentangle the role of geomorphology and climate changes on their diversification), with particular focus on dispersal events among major dry regions in Africa and between Africa, the Arabian Peninsula and the Eastern Mediterranean; (4) to estimate the species richness of the genus by applying of the phylogenetic species concept to identify the genetic groups and geographical regions that are worth further integrative taxonomic studies.

## Results

### Phylogeny, species delimitation, and distribution of genetic variability of Acomys

**Fig. 1:**
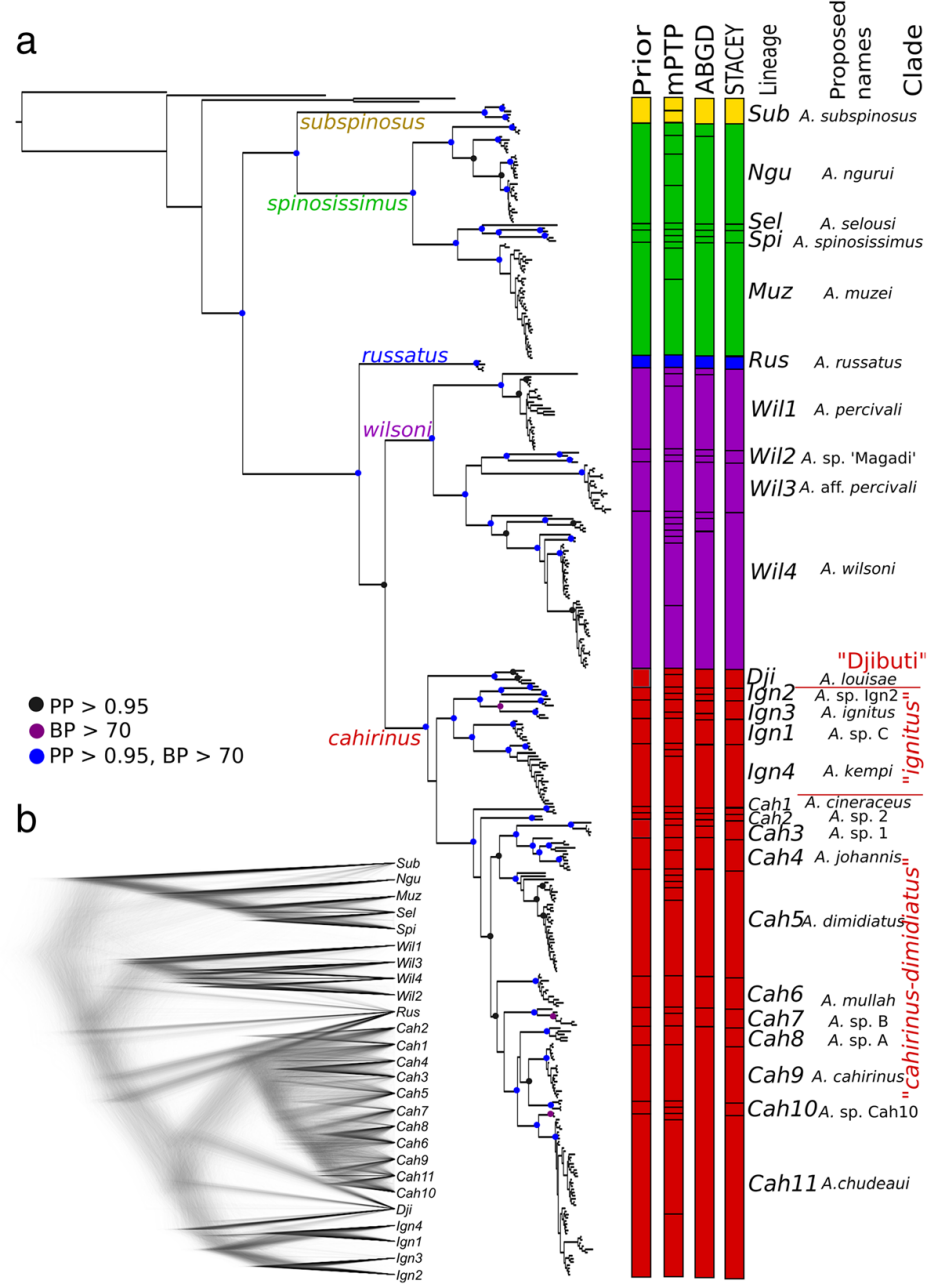
Multilocus phylogeny of the genus *Acomys.* **a** Bayesian phylogeny of the concatenated multi-locus matrix calculated in MrBayes. The support from Bayesian analysis in MrBayes (posterior probability, PP) and maximum likelihood analysis in RAxML (bootstrap probability, BP) is indicated by different colours on the nodes (black PP > 0.95, BP < 70; violet PP < 0.95, BP > 70; blue PP > 0.95, BP > 70). Five main *Acomys* groups (*subspinosus*, *spinosissimus*, *russatus*, *wilsoni* and *cahirinus*) are shown by different colours. The results of four different species delimitation approaches (“by-eye” prior; two delimitation approaches based on mtDNA: mPTP, ABGD; and a multilocus species delimitation in STACEY - see more details in the text) are shown in columns on the right, where individual “species” are separated by black lines. Additional information for 26 delimited taxa are provided, abbreviation of the lineages, and previously used taxonomic assignments. **b** DensiTree cloudogram of coalescent species trees from STACEY (for MCC species tree with PP see Additional file 4)

Both Bayesian inference (BI) and Maximum Likelihood (ML) analyses of concatenated multilocus data provided very similar phylogenetic reconstructions and revealed five major groups that we will hereafter call *subspinosus*, *spinosissimus*, *russatus*, *wilsoni* and *cahirinus* (Fig. 1a). If the number of nodes that are supported by PP ≥ 0.95 or BP ≥ 70 are considered, BI analyses yielded a slightly more robust topology (79 supported nodes; topology shown at Fig. 1a) compared to the ML tree (64 supported nodes; topology not shown). Five major groups are also supported in separate mitochondrial (not shown) and nuclear (Additional files 2 and 3) gene trees.

**Fig. 2:**
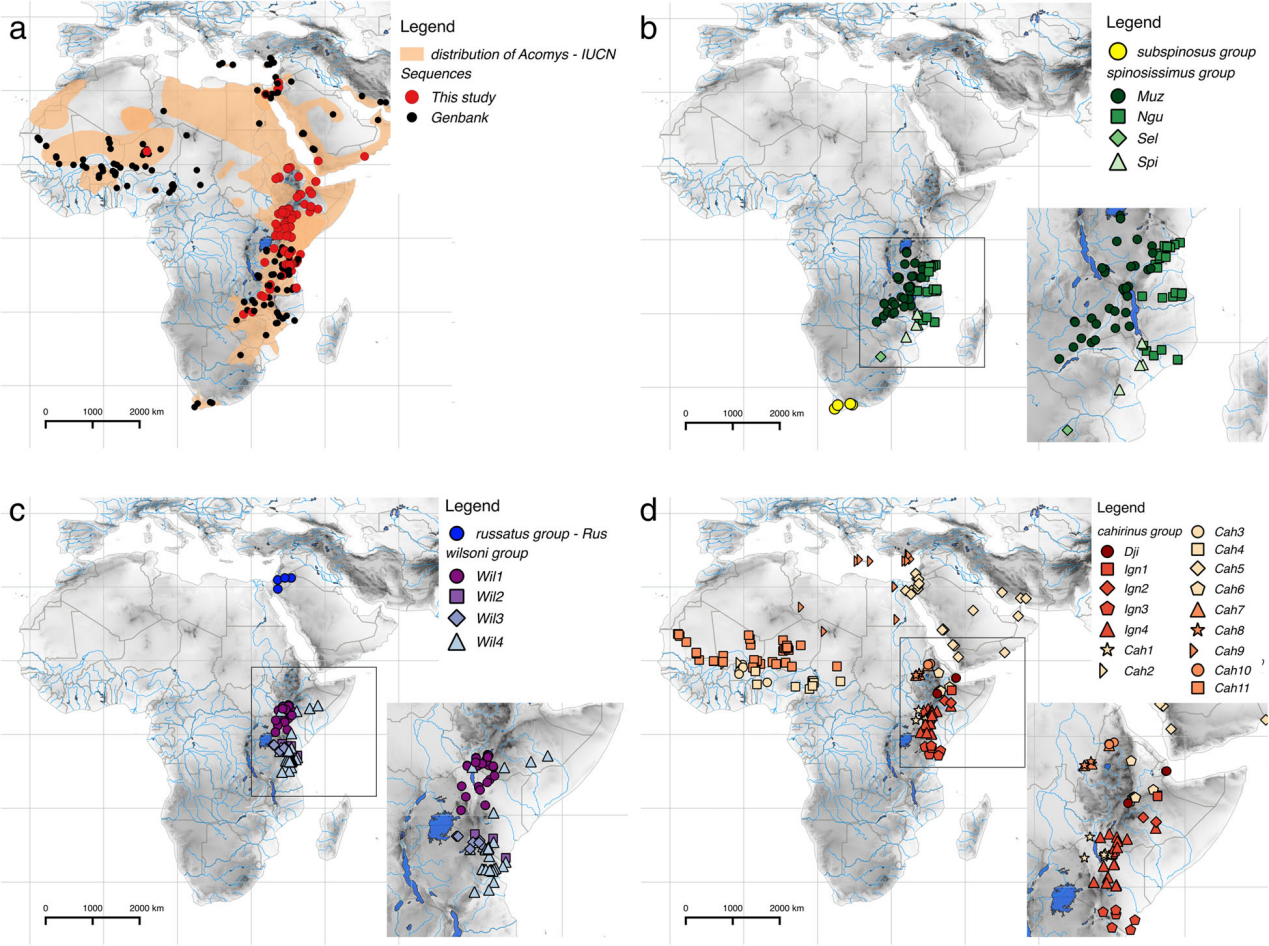
Analysed samples and the distribution of genetic variability in the genus *Acomys*. **a** Geographical distribution of the genus *Acomys* according IUCN (orange background); the origin of newly genotyped individuals is shown by red circles, while georeferenced sequences from GenBank are shown by black circles; **b** distribution of genetic lineages in the *subspinosus* and *spinosissimus* groups; **c** distribution of genetic lineages in the *russatus* and *wilsoni* groups; **d** distribution of genetic lineages in the *cahirinus* group

Three of these major groups (*spinosissimus*, *wilsoni*, and *cahirinus* groups) are further sub-structured to phylogenetic lineages (Fig. 1, Additional files 2 and 3) with predominantly parapatric distributions (Fig. 2). Based on the topology and the shape of the phylogenetic trees and the geographical distribution of genetic variability, we identified 26 distinct genetic lineages as our prior candidate species for next steps of genetic species delimitation (Fig. 1a). Their mutual relationships are relatively well resolved (with three well-defined clades: “Djibuti”, “*ignitus*” and “*cahirinus*-*dimidiatus*”, in the *cahirinus* group), with the important exception of lineages *Cah1-Cah11* representing probably a rapid radiation of the “*cahirinus*-*dimidiatus*” clade (Fig. 1a). The multispecies coalescent species tree from Species Tree And Classification Estimation, Yarely (STACEY) also revealed very similar topology to that reconstructed from the concatenated super-matrix (Fig. 1b, Additional file 4). The main differences are in unresolved positions of the *russatus* group and the “Djibuti” clade and a weakly resolved topology within the three major groups (*spinosissimus*, *wilsoni*, *cahirinus*; Additional file 4).

Species discovery approaches based on mitochondrial cytochrome *b* gene (*CYTB*) variability split the genus *Acomys* into 57 putative species in multi-rate Poisson Tree Process (mPTP) and 32 putative species in Automatic Barcode Gap Discovery (ABGD; Fig. 1), but ABGD did not find any clear gap between intra- and interspecific distances (“barcoding gap”; not shown). On the other hand, results from multispecies coalescent analysis in STACEY supported all 26 a priori defined species as separate gene pools (Fig. 2a). Taking into account the fact that multispecies coalescent does not statistically distinguish structure associated with population isolation vs. species boundaries [49], we will therefore use the term “species” for genetically distinct lineages or molecular operational taxonomic units (MOTUs).

The *subspinosus* group with only one lineage (*Sub*) is limited to South Africa (Fig. 2b). The separate species status was confirmed by all species delimitation analyses, and mPTP even suggested two different mitochondrial sublineages as two separate species (Fig. 1a). Mean intraspecific genetic distance is 1.5%, the distance to the nearest lineage is 21.3% (Table 1).

**Table 1:** Genetic distances calculated from BI phylogenetic tree by the Species Delimitation algorithm in Geneious. Intraspecific distances and interspecific distances from the nearest lineage in percents (%)

Group	Lineage	Nearest lineage	Intra Dist	Inter Dist - Closest
*subspinosus*	*Sub*	*Ngu*	1.5	21.3
*spinosissimus*	*Ngu*	*Muz*	2.9	10.7
	*Sel*	*Spi*	0.0	7.1
	*Spi*	*Sel*	3.2	7.1
	*Muz*	*Spi*	1.1	8.0
*russatus*	*Rus*	*Ign1*	0.5	14.4
*wilsoni*	*Wil1*	*Wil2*	1.5	12.3
	*Wil2*	*Wil3*	4.7	10.5
	*Wil3*	*Wil2*	1.0	10.5
	*Wil4*	*Wil2*	3.1	9.6
*cahirinus*	*Dji*	*Ign1*	2.2	9.6
	*Ign2*	*Ign3*	2.9	6.4
	*Ign3*	*Ign2*	2.9	6.4
	*Ign1*	*Ign4*	0.7	5.9
	*Ign4*	*Ign1*	1.6	5.9
	*Cah1*	*Cah2*	0.6	7.5
	*Cah2*	*Cah6*	0.8	6.5
	*Cah3*	*Cah4*	0.8	6.1
	*Cah4*	*Cah5*	2.1	6.0
	*Cah5*	*Cah4*	1.4	6.0
	*Cah6*	*Cah9*	0.8	5.5
	*Cah7*	*Cah9*	1.3	5.5
	*Cah8*	*Cah9*	1.2	4.0
	*Cah10*	*Cah9*	0.6	3.0
	*Cah9*	*Cah10*	0.6	3.0
	*Cah11*	*Cah9*	1.2	4.5

The *spinosissimus* group inhabits the eastern part of the Zambezian bioregion (Fig. 2b). The STACEY approach confirmed four distinct species (*Muz, Ngu, Spi, Sel*), while mPTP and ABGD suggested 10 and six species, respectively (Fig. 1a). The intraspecific *CYTB* distances in four species range from 1.1 to 3.2% (excluding *Sel*, where only a single sequence was available). Interspecific distances to the nearest neighbour are 7.1-27.0% (Table 1).

The distribution of sequenced samples from the *russatus* group is restricted to arid regions of the Levant (Jordan, Israel; Fig. 2c). A single species (*Rus*) was supported by all species delimitation analyses. The mean intraspecific distance is low (0.5%), while interspecific distance to the nearest neighbour is 14.4% (Table 1).

The *wilsoni* group is divided into four well supported lineages, suggested as separate species by STACEY, and they are predominately distributed in the Somali-Masai savanna (Fig. 2c): *Wil1* lives on both sides of the Great Rift Valley (GRV) in Kenya and Ethiopia, *Wil2* and *Wil3* are two lineages with parapatric distribution in southern Kenya and northern Tanzania, and, finally, *Wil4* was found east of GRV from north-eastern Tanzania to southern Ethiopia, where it overlaps with *Wil1.* The mPTP split the *wilsoni* group into 13 putative species, and ABGD into eight species. The intraspecific distances of four STACEY species ranged from 1.0 to 4.7%, interspecific distances among them are from 10.5 to 12.3% (Table 1).

The highest genetic diversity was found within the *cahirinus* group (16 lineages delimited as species by STACEY). The group is composed of three significantly supported clades (with unresolved relationships among them), distributed parapatrically, with only small overlap (Fig. 2d): (i) the clade “Djibuti” with a single species (*Dji*) recorded from Djibuti and Dera National Park in Ethiopia, geographically neighbouring the Ethiopian Afar province; (ii) the clade “*ignitus*” distributed south-east of GRV with four lineages (*Ign1-Ign4*); (iii) the clade “*cahirinus*-*dimidiatus*” widespread north-west of GRV, including Sahel and Sudanian savanna, eastern Mediterranean, Middle East and Arabian peninsula, with 11 species (*Cah1-Cah11*) delimited by STACEY (Fig. 1). Intraspecific distances within the *cahirinus* group range from 0.6 to 2.9%, interspecific from 3.0 to 9.6% (Table 1).

### Historical biogeography and divergence dating

**Fig. 3:**
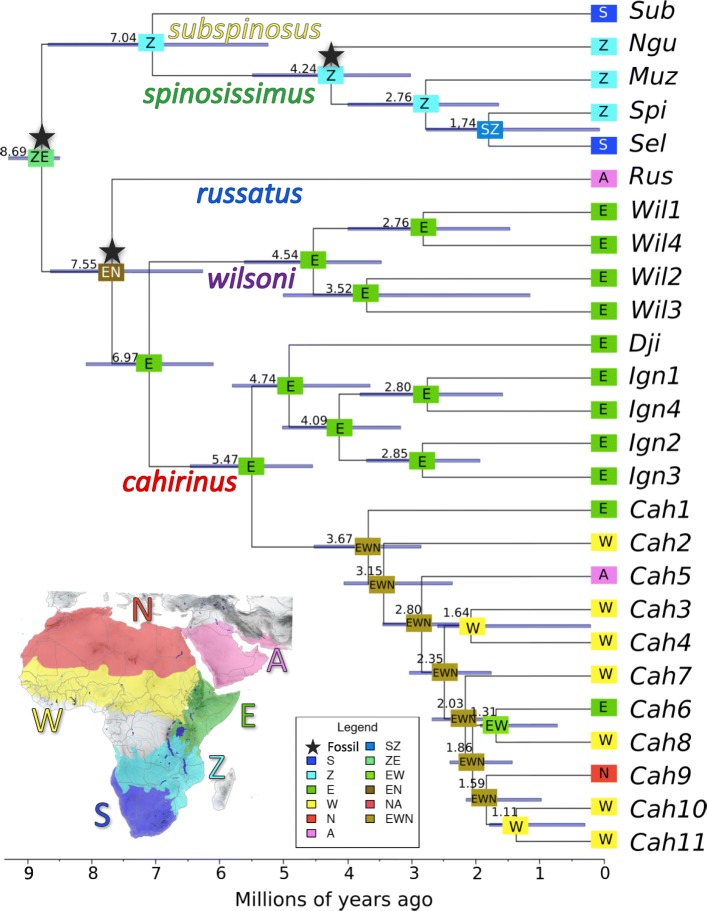
Divergence dating and the reconstruction of historical biogeography. Numbers on the nodes represent medians of estimated divergence date, and the horizonal bars show 95% highest posterior density of these estimates. Stars indicate the positions of fossil constrains used for the calibration of molecular clock (see Table 3 for more details). Different colours on the nodes represent reconstructed ancestral regions for each clade, according to the map in the frame: South Africa region (S), Zambezian region (Z), Somali region (E), Sudanian region (W), Sahara region (N), Arabian region (A)

Based on the Dispersal-Extinction Cladogenesis model and the time-calibrated tree, the TMRCA of the genus *Acomys* is dated to 8.69 Ma (95% HPD = 8.51–9.29 Ma). The ancestral area is predicted to be in Eastern Africa, but it is not resolved whether in the Zambezian or the Somali region (Fig. 3). The first split in the Late Miocene separated southern groups (*subspinosus + spinosissimus*) from northern groups (*russatus + wilsoni + cahirinus*). Around 7.04 Ma (95% HPD = 5.25–8.67 Ma) the ancestor of the *subspinosus* group in South Africa diverged from the ancestor of the *spinosissimus* group in the Zambezian region, where the latter group started to diverge around 4.24 Ma (95% HPD = 3.02–5.48 Ma). The ancestor of the *russatus* group in Arabian region separated from *Acomys* in Somali region around 7.55 Ma (95% HPD = 6.27–8.64 Ma). The split between *cahirinus* and *wilsoni* groups occurred in Somali region around 6.97 Ma (HPD = 6.10–8.08 Ma). The *wilsoni* group started to diversify around 4.54 Ma (95% HPD = 3.48–5.61 Ma), while the first split in the *cahirinus* group is dated to 5.47 Ma (95% HPD = 4.55–6.46 Ma), in both cases the beginning of radiation is predicted in the Somali-Masai savanna. Subsequent splits in the *cahirinus* group occurred around 5 Ma, when the ancestors of clades “Djibuti”, “*ignitus*” and “*cahirinus*-*dimidiatus*” diverged most likely in Eastern Africa. The biogeographic history of the “*cahirinus*-*dimidiatus*” clade was more complex. While *Cah1* stayed in Somali region, the ancestor of lineages *Cah2-Cah11* likely dispersed to the north-west (especially Sudanian savanna), where most genetic lineages currently occur. The results suggest one Pleistocene migration back to the Somali region (*Cah6* in Afar region, ~ 1.31 Ma) and two independent dispersals to the north, either to the Arabian region (*Cah5* dated at 2.80 Ma) and to the Sahara region (*Cah9* dated at 1.59 Ma).

### Species distribution modelling

**Fig. 4:**
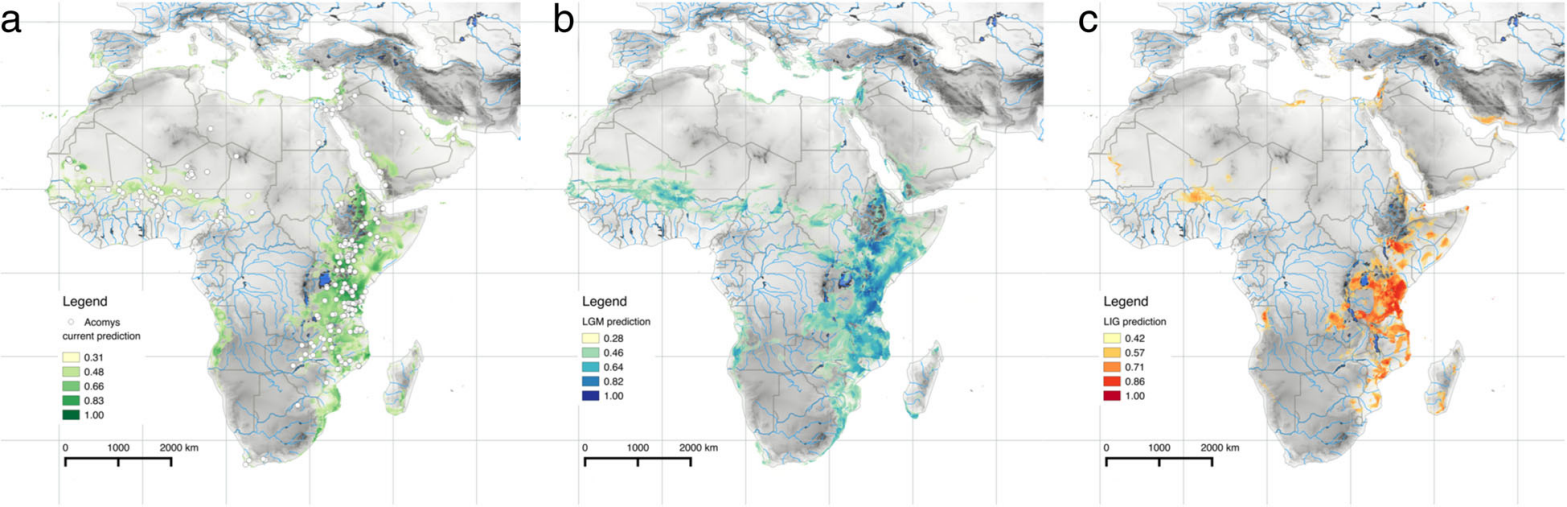
The probability of *Acomys* occurrence based on the MaxEnt modelling of bioclimatic niches. More intensive colour indicates higher probability of suitable conditions. **a** The model for the present; white dots indicate the sampled localities; **b** prediction for the last glacial maximum (21 ka); **c** prediction for the last interglacial period (120–140 ka)

The bioclimatic MaxEnt model for the present shows that the sampling in this study covers almost the complete range of suitable climatic conditions for genus *Acomys* (Fig. 4a). The most important variables predicting the modelled geographic distribution of spiny mice were the annual range of temperature and annual precipitation. The predicted distribution is relatively continuous in eastern Africa, mainly in the Somali-Masai and eastern Zambezian savanna. On the contrary, climatically unsuitable are moist mountains in Ethiopia, Kenya and Albertine Rift, as well as very arid regions of Horn of Africa and Masai xeric scrublands in north-eastern Kenya. The belt of suitable climatic conditions occurs in West Africa, on the boundary between Sudanian savanna and Sahel, as well as along Mediterranean sea and Arabian Peninsula and southern Iran. Isolated suitable areas are predicted in western Angola and northern Namibia, despite the absence of present-day occurrence of *Acomys* species. The model for LGM predicts very similar distribution of spiny mice, with more continuous belt of suitable conditions in Sudanian region (Fig. 4b). The predicted distribution during LIG was more fragmented, with highly suitable conditions in southern Ethiopia, Kenya and Tanzania, and isolated patches in the Horn of Africa, southern part of the Sahara desert, and Mozambique (Fig. 4c). Altogether, it seems very likely that the climatic conditions in the core of the present day distribution, in open seasonally dry habitats in East Africa, were apparently favourable at least during the last glacial cycle.

## Discussion

Spiny mice of the genus *Acomys* represent a speciose group of rodents, widely distributed in seasonally dry savanna of Africa, Arabia and Middle East. They often are a dominant component of small mammal assemblages and in some habitats, e.g. in rocky outcrops, they can be even the only rodents captured (our unpubl. data). They were able to colonize wide spectrum of non-forested habitats, from miombo woodlands to rocks in the middle of Sahara, from the sea coast up to 2500 m above sea level (a.s.l.) [e.g.*,* 36]. Despite *Acomys* abundance and practical importance (e.g.*,* as model taxa in behavioural or biomedical research; [50]), the knowledge of their evolutionary history, taxonomy and biogeography has been very limited and biased to particular regions or intrageneric clades [28, 30, 31, 33, 41, 43, 45]. Here we compiled the largest multilocus genetic dataset to date for the genus *Acomys* (genotypes of 699 individuals from more than 280 localities covering a majority of the distribution of this genus), reconstructed phylogenetic relationships, described biogeographical patterns and evolutionary history, and estimated the spiny mice species diversity.

### Phylogeny and biogeographical patterns in the genus *Acomys*

Phylogenetic analysis revealed clear evidence for the existence of five major groups, *subspinosus, spinosissimus, russatus, wilsoni* and *cahirinus* (Figs. 1 and 2, Additional files 2 and 3), which diverged in the late Miocene (Fig. 3). For the first time we provide nuclear genetic data for the *subspinosus* group and we refer its sister position with the *spinosissimus* group, which has never been confirmed [31, 41, 45]. This sister relationship is supported also by the fact that *subspinosus* and *spinosissimus* groups share the same triplicate-type X chromosome that is very rare among mammals [51]. For the first time we also sequenced the nuclear markers of the *russatus* group, but its phylogenetic relationships with the *wilsoni* and *cahirinus* groups remained unresolved (Fig. 1), which suggests fast divergence in the late Miocene (see similar results in [45]). Using multilocus genetic data, we unequivocally identified that the first split within the genus occurred between south-eastern and eastern Africa, i.e. (*subspinosus + spinosissimus*) vs. (*russatus + wilsoni + cahirinus*) (Fig. 3). Further diversification of the five major groups is dated to Plio-Pleistocene and, interestingly, the diversification rate within them is very unequal. While *subspinosus* and *russatus* remained monotypic at the margins of *Acomys* distribution (in the Cape Region and Middle East, respectively), *spinosissimus* and *wilsoni* clades diversified by a comparable rate in Eastern Africa, either in Zambezian or Somali-Masai savannas. The most intensive spatial and cladogenetic expansion occurred in the *cahirinus* group that colonized large areas of Africa, Arabia and eastern Mediterranean region, even if the most genetic variation is still observed in eastern Africa.

Our phylogenetic analysis revealed at least three independent “out-of-Africa” events, differing significantly by the date of divergence from their African counterparts. The oldest disperser was the ancestor of the *russatus* group, currently distributed only in the Sinai Peninsula and the Middle East, which split from the ancestors of the *wilsoni* and *cahirinus* groups already in late Miocene (Fig. 3, see also [30]). Much more recently, at the beginning of Pleistocene, the ancestors of *Cah5* colonized the Arabian Peninsula (and South Iran). It is unclear whether it was through the Bab al-Mandab connection between Africa and Yemen [33, 52] or through Sinai and around the Red Sea coastline [53]. Lastly, the western Mediterranean islands and south coast of Turkey were colonized during Antiquity by possibly commensal populations from the lineage *Cah9*, likely from Egypt [31, 33, 54].

Spiny mice of the *cahirinus* group (specifically “*cahirinus-dimidiatus*” clade) are widely distributed also in the Sudanian savanna. Results of our biogeographical analysis suggest multiple colonization and diversification waves, directed usually from eastern to western Africa. The first split between *Cah1* (which stayed restricted in Somali region along the Lake Turkana) and remaining lineages occurred in the late Pliocene/early Pleistocene. The ancestors of lineages *Cah2*, *Cah 3*, and *Cah4* diverged in southern (more humid) part of Sudanian savanna probably simultaneously with the colonization of Arabian Peninsula by *Cah5.* Much later (*ca.* 1–1.5 Mya), the split between northern (*Cah9*) and southern (*Cah10* and *Cah11*) resulted in current diversity in large arid areas in northern part of the Sudanian region, Sahel and Sahara desert. At the same time the Ethiopian highlands caused the diversification of *Cah8* (living in easternmost Sudanian region) and *Cah6* (currently limited to Afar triangle). Faunal exchange between Somali-Masai and Sudanian savanna in the Plio-Pleistocene have been described also in other rodents and very often the oldest lineages of Sudanian taxa are found in Eastern Africa [55–57], which well corresponds with the pattern found in spiny mice.

The current distribution of genetic diversity of *Acomys* is affected by major biogeographic divides, represented by (1) extremely dry open habitats, (2) mountain blocks and (3) large water bodies, e.g. Rift Valley lakes or rivers. Even if the spiny mice are considered as typical inhabitants of (semi-)arid environments [58], they avoid extremely dry habitats. Distribution modelling based on bioclimatic data (Fig. 4) suggests low suitability of habitats in most of Saharan and Arabian deserts, as well as in Masai xeric bushland and shrublands in northernmost Kenya and Horn of Africa. However, even in such habitats the spiny mice can occur, but they have very patchy distribution with isolated populations in rocky areas, considered as remains of more continuous distribution in past humid periods, when savanna-like habitats prevailed [43, 59–63]. Sahara desert seems to work as a barrier between clades *Cah9* and *Cah11* (albeit the data are missing e.g. from southern Algeria).

The expansion of forests during interglacials [64, 65], known as Pleistocene breathing model [66], repeatedly fragmented savanna biome, especially in eastern Africa. The forested mountain blocks thus formed an important biogeographical divide for savanna-dwelling organisms and supported allopatric diversification in this nowadays relatively continuous ecosystem [67]. In agreement with this hypothesis, we can see the coincidence of genetic structure in the *spinosissimus* group with Eastern Arc Mountains (EAM) and Southern Rift Mountains (SRM). These mountain ranges, even if permeable today for taxa living in open habitats, clearly separate *Ngu* and *Muz* lineages (Fig. 2b; see more details in [44]). Similar structure was recently observed in numerous other taxa of sympatric murid and bathyergid rodents living in open habitats of Zambezian region [67–70]. Similarly, the mountains in north-eastern Tanzania (Kilimanjaro, Pare, Usambara) currently delimit distribution for some *Acomys* lineages (*Wil2* vs. *Wil3* or *cahirinus* vs. *spinosissimus* groups), and, again, this pattern was found in other savanna rodents (e.g. *Saccostomus* [71], *Gerbilliscus* [72]). Further to the north, Kenyan highlands could have worked in similar way as they seem to limit distribution of several *Acomys* lineages (*Wil1* vs. *Wil2 + Wil3, Ign3* vs. *Ign4*). Lastly, Ethiopian Highlands, delimit distributions of lineages especially in the *cahirinus* group. For example lineages *Dji* and *Cah6* are restricted to the Afar province in north-eastern Ethiopia (see also very similar pattern in gerbils of the genus *Gerbilliscus*, often sympatric with spiny mice; [72]).

Rivers and other more or less linear water bodies (e.g.*,* contemporary rift lakes, or palaeolakes; [73]) are known to play an important role in shaping genetic structure and diversification patterns in organisms living in open habitats. For example the Zambezi-Kafue river system delimits southern border of distribution of *Ngu* and *Muz* lineages (Fig. 2b; [44]) and have been implicated in forming genetic diversity in savanna-dwelling organisms as diverse as killifishes [74], gerbils [75], African pouched mouse [71], baboons [76] and several species of antelopes [77]. The largest river in Sudanian savanna is the Niger River, which makes a border between *Cah11* and other West African *Acomys* lineages, is also dominant biogeographical divide in many other rodents (e.g.*,* [43, 55, 67, 78]). On the other hand, the Nile Valley seems to serve rather as a suitable migration corridor for northward spreading of savanna taxa from eastern Africa (see [56] for grass rats, or [79] for shrews). In *Acomys*, it very likely allowed the lineages *Cah5, Cah9* and *Rus* to colonize the northern Africa and Arabian Peninsula. Trauth et al. [73] suggested that during the humid periods of Pleistocene, the bottom of GRV was filled by water (much more than today), and the so-called palaeolakes formed an important biogeographic barrier causing allopatric diversification or even speciation. The phylogeographic pattern concordant with this hypothesis was recently described in Eastern African gerbils [72] and the role of GRV on genetic structure is visible also in *Acomys*. For example, *Cah1* is separated from “*ignitus*” clade by GRV and, similarly, more records of *Wil1* occur west of GRV, while other lineages of the *wilsoni* group were found predominantly east of GRV.

### Evolutionary scenario of the genus *Acomys* − interplay of geomorphology and climatic changes

The origin of *Acomys* is dated in late Miocene, which is in concordance with the first occurrence of savannas that appeared as a result of rifting activity in eastern Africa and climatic changes [4]. Spiny mice belong to the subfamily Deomyinae, whose other members occur mostly in forest or forest margins (i.e. genera *Deomys* and *Lophuromys*) or moist savanna (*Uranomys*) [16]. The phylogenetic relationships among genera of Deomyinae are not sufficiently resolved [46, 80, 81], but it seems likely that the ancestor of *Acomys* colonized dry open habitats early after their late Miocene appearance profiting from empty niches in this ecosystem. Barome et al. [31] proposed the origin of *Acomys* ca. 13.7 Ma in East Africa or in South Africa, while Alhajeri et al. [46] placed it more generally to sub-Saharan Africa at 10 Ma. Other authors [40, 82] suggested the origin for the genus in Eastern Africa, mainly in the Ethiopian region [82]. Our historical biogeography reconstruction is in partial agreement with previous studies as the origin of the genus is placed either in Somali and/or Zambezian regions (Fig. 3), i.e. the regions with the highest contemporary genetic diversity of spiny mice. Even if the oldest fossil records of *Acomys* ancestors are found in Zambezian savanna (†*Preacomys griffini* and †*Preacomys karsticus* ca. 9 Ma from Namibia; [83]), they occurred in the late Miocene also in East Africa (†*Preacomys kikiae* 8.5 Ma from Chorora, Ethiopia; [84]).

Periods of warm humid climate in Late Miocene caused the last occurrence of coast-to-coast belt of tropical forest, which is evidenced by phylogenetic analyses of plants and animals living in nowadays fragmented forests of Congo basin and eastern African montane and coastal forests [85, 86]. This continuous forest was likely one of the most important factors in early evolution of savanna inhabitants, because it separated northern (= Somali-Masai) and southern (= Zambezian) savannas. In *Acomys*, this resulted in allopatric divergence between the ancestor of *subspinosus + spinosissimus* group in the Zambezian savanna and the ancestor of *wilsoni + russatus + cahirinus* in the Somali-Masai savanna (estimated in this study at 8.7 Ma). The same geographical and temporal pattern, i.e. late Miocene split of northern and southern taxa, was observed also in other savanna mammals, e.g. gerbils [57], pouched mice [71], several genera of antellopes [87], warthogs [88] and giraffes [89, 90] .

Later on, but still in Late Miocene, the evolution of savanna’s fauna was significantly influenced mainly by the Messinian salinity crisis (MSC, [91]), dated to 6.0–5.3 Ma. Very little is known about the effect of the MSC on eastern African climate [6], but it is generally held that overall aridification at the Miocene/Pliocene boundary promoted the expansion of very dry habitats [92]. Inhospitable very arid (desert) areas in north-eastern Africa expected at MSC period thus likely interrupted the connection of *Acomys* populations between Somali-Masai and eastern Mediterranean area. This period corresponds to the split of the *russatus* group, which remained effectively isolated in the north.

Plio-Pleistocene period (starting 5.3 Ma) is characterized by intensive climatic oscillations. There are several well-known climatic transitions, like the intensification of Northern Hemisphere glaciation (iNHG; 3.2–2.5 Ma, [93, 94]), the development of the Walker circulation (2.0–1.7 Ma; [95]) and the early-middle Pleistocene transition (1.2–0.8 Ma; [96]). These periods of pronounced climate variability significantly affected the distribution of forests, palaeolakes and savannas [97]. For example during more humid periods the currently fragmented montane forests in Eastern Arc Mountains and Kenyan highlands probably expanded into lower altitudes, became more continuous and formed significant barriers to gene flow for taxa living in open dry habitats [67, 71, 72, 98]. The bottom of GRV was filled by the palaeolakes, which prevented gene flow of savanna-dwelling species, leading to diversification, or even speciation [73]. Because of wide confidence intervals of our estimates of divergence times, it is not possible to link particular splits to specific climatic events. However, it is highly probable that a majority of current genetic diversity in *spinosissimus*, *wilsoni* and *cahirinus* groups is a result of repeated fragmentation of savannas in Plio-Pleistocene, caused by climatic changes. This is further supported by parapatric distribution of lineages within major clades, where the distribution borders often correspond to predicted barriers of the gene flow (i.e. too arid or forested areas, and palaeolakes).

### Species richness of *Acomys* − the need of further integrative taxonomic studies

The number of species crucially depends on adopted species concept. Rapidly increasing amount of genetic data now allows to apply the so-called integrative taxonomic approach, which usually complements the widely used typological or biological species concepts by genetic [99] and/or phylogenetic [100] species delimitations. The taxonomy of *Acomys* has been unresolved since the second half of the twentieth century. In Additional file 1 we listed several taxonomic alternatives, based mostly on morphological traits, used recently for the genus *Acomys*. The most comprehensive list was provided by Ellerman [34] with 25 species and 17 subspecies. On the other extreme, Setzer [38] recognized only five species that partly correspond to major genetic clades recovered in our study: *A. cahirinus* (7 subspecies), *A. dimidiatus* (12 subspecies), *A. russatus* (one subspecies), *A. spinosissimus* (one subspecies) and *A. subspinosus* (9 subspecies, including *A. wilsoni*). Widely accepted list of Musser and Carleton [37] contained 18 species. Monadjem et al. [36] lists 15 species in sub-Saharan Africa, including three newly delimited species in the *spinosissimus* group [45]. The most recent and comprehensive Handbook of the Mammals of the World [16] listed 21 species and 12 subspecies.

We used several genetic species delimitation methods and their estimates of spiny mice species richness differ significantly from 57 species (mPTP) to 26 species (STACEY as well as our prior delimitation based on geographical distribution of genetic diversity). The species delimitation based only on mitochondrial markers (ABGD and mPTP) have the tendency to overestimate the number of revealed species and they often identify as separate species also genetic lineages that are traditionally considered as intraspecific phlyogeographic structure. For example ABGD revealed four species within *Ngu* lineage, while Petružela et al. [44] recently showed on multi-locus dataset that they represent only phylogeographic structure of *A. ngurui*. Below we use the most conservative estimate (26 MOTUs, here considered as “genetic lineages” and named according Fig. 1a) and compare these species delimitations with previous taxonomic work. Multi-species coalescent approaches to species delimitation (like STACEY) in fact diagnose the genetic structure, with no distinction between structure due to populations or due to species [49]. Therefore, the aim of the following part is not to perform a formal taxonomic revision, but to show the genetic clades and geographic regions where further integrative taxonomic analyses (employing combination of genetic, morphological, ecological and other data) could lead either to new descriptions or synonymization of *Acomys* taxa.

#### The subspinosus group

This group is monotypic and contains a single lineage *Sub*.

*(1) Sub*

*Distribution:* South Africa (Cape Province).

*Available name:* *Acomys subspinosus* (Waterhouse, 1838).

*Type locality:* Western Cape Province, Cape of Good Hope, South Africa.

*Karyotype:* 2n = 64, NF = 70 [101, 102].

*Additional information:* Based on its unique dental and skull morphology, *A. subspinosus* has been placed in its own subgenus *Subacomys* with an “ancestral” karyotype (2n = 64, NF = 70; [51, 101–104]). Its separation from other *Acomys* was also indicated by phylogenetic analyses of *CYTB* [31, 32, 45]. Using for the first time the combination of mitochondrial and nuclear markers, we unequivocally showed its sister relationship with *spinosissimus* group, i.e. it does not represent the first cladogenetic split of *Acomys*. As a consequence, the validity of the subgenus *Subacomys* (mentioned erroneously as *Preacomys* in Denys et al. [16]) is questionable.

#### The *spinosissimus* group

This strongly supported monophyletic group has been revised repeatedly [41, 44, 45] and four genetic lineages were distinguished and named. However, the genetic data from the southern part of its distribution are still very limited and especially the taxonomy and distribution of *A. selousi* and *A. spinosissimus* should be further explored.

*(2) Ngu*

*Distribution:* Lineage *Ngu* is distributed in three well supported parapatric sublineages from Tanzania (East of EAM) to central Mozambique (north of the Zambezi River; [44]).

*Available name:* *Acomys ngurui* Verheyen et al., 2011.

*Type locality:* Nguru Ya Ndege, Tanzania.

*Karyotype:* 2n = 60, NFa = 68 [45].

*Additional information:* This species is very similar to *A. muzei* from which it differs by relatively shorter tail and non-overlapping distribution [44, 45]. Barome et al. [41] reported it as *A.* cf. *selousi* from Berega. Three genetically distinct sublineages probably represent intraspecific variation [44].

*(3) Sel*

*Distribution:* Northern part of South Africa, only one genetically confirmed locality from the Kruger National Park [45]. Northern limits of its distribution are not fully resolved.

*Available name:* *Acomys selousi* De Winton, 1896.

*Type locality:* Essex Farm, Zimbabwe.

*Karyotype:* 2n = 58–62, NFa = 68 [101, 102] .

*Additional information:* According some authors [35, 37, 38]; *A. transvaalensis* and *A. selousi* are synonyms of *A. spinosissimus*. Here we follow the view of Verheyen et al. [45] and Monadjem et al. [36] and consider this lineage as a separate species, but further taxonomic investigation of the *spinosissimus* group in South African region is required.

*(4) Spi*

*Distribution:* Mozambique, Zimbabwe, southern Malawi.

*Available name:* *Acomys spinosissimus* Peters, 1852.

*Type locality:* Tette and Buio, Mozambique.

*Karyotype:* 2n = 60, NFa = 68 [101].

*Additional information:* We follow the view of Verheyen et al. [45] and Monadjem et al. [36] and include only populations from central Mozambique and southern Malawi into this species. Petružela et al. [44] recently showed that its distribution north of the Zambezi River is much more restricted compared to maps in Monadjem et al. [36] and the two well distinct genetic sublineages of this species seem to be separated by the Zambezi river.

*(5) Muz*

*Distribution:* Central and western Tanzania, Malawi (west of the Lake Malawi), Zambia.

*Available name:* *Acomys muzei* Verheyen et al., 2011.

*Type locality:* Muze, Tanzania.

*Karyotype:* 2n = 58–62, NFa = 68 [45].

*Additional information:* Recent analyses showed that the populations in Malawi and Zambia (reported as *A. spinosissimus* in Monadjem et al. [36]) belong to this species [44]. Further, the highest genetic diversity of the species was recorded west of the Lake Malawi, while Tanzanian populations represent only relatively recent colonization event [44].

#### The *russatus* group

The *russatus* group is monotypic with only one lineage *Rus*. The phylogenetic relationships with its sister groups *cahirinus* and *wilsoni* are not fully resolved (Fig. 1).

*(6) Rus*

*Distribution:* Egypt (separate subspecies *aegyptiacus* was described in Eastern Desert), Sinai, Jordan, Israel, Saudi Arabia, Yemen and Oman. Genotyped material in this study originates only from Jordan and Israel.

*Available name:* *Acomys russatus* (Wagner, 1840).

*Type locality:* Sinai, Egypt.

*Karyotype:* 2n = 66, NF ≥ 66 [40].

*Additional information:* Denys et al. [51] referred that *A. russatus* is very distinctive in its molar morphology and chromosomal traits and previous *CYTB* [31, 41, 45] as well as our multilocus genetic analyses showed that it is not closely associated with any other *Acomys* taxon. *Acomys russatus* and *A. dimidiatus* (= *Cah5* in this study) can live in sympatry, and their differences in ecology, physiology, and activity patterns (especially in Israel) have been extensively documented (see references in [105]).

#### The wilsoni group

This group is well supported in all phylogenetic analyses (Fig. 1 in this study, [31, 41]), but its relationships with *russatus* and *cahirinus* groups are not completely resolved. Distribution of the *wilsoni* group is limited to the Somali region [sensu 3]. Based on multilocus species delimitation we recognize four genetic lineages, but the species limits must be further investigated.

*(7) Wil1*

*Distribution:* South Ethiopia, Kenya (along GRV).

*Available name:* *Acomys percivali* Dollman, 1911.

*Type locality:* Chanler Fall, Nyiro, Kenya.

*Karyotype:* 2n = 36 and NF = 68 [40].

*Additional information:* Phylogenetically the most distinct MOTU within the *wilsoni* group (Fig. 1). It was not included in previous phylogenetic studies. Distribution of *A. percivali* reported by Monadjem et al. [36] and Denys et al. [16] is very similar to that of *Wil1*. Janecek et al. [103] regarded *A. percivali* as the species genetically most closely related to *A. wilsoni* (*= Wil4*)*.* Both clades were found sympatric at several localities in southern Ethiopia, where they can be distinguished by external morphology (our unpublished data) and karyotypes [16].

*(8) Wil2*

*Distribution:* Southern Kenya.

*Available name:* None. Based on Barome et al. [31], we use in Fig. 1 the name *A.* sp. ‘Magadi’.

*Type locality:* Not relevant.

*Karyotype:* Not known.

*Additional information:* Known only from three localities from southern Kenya, all of them reported by Barome et al. [31]. They mentioned this MOTU as two different species, *A*. sp. ‘Magadi’ and *A. wilsoni*, and this structure was reflected also by mPTP and ABGD analyses in our study. The conspecificity with *Wil3* and/or *Wil4* are plausible hypotheses and should be tested.

*(9) Wil3*

*Distribution:* NW Tanzania, southern Kenya, most localities in the bottom of GRV.

*Available name:* None. Based on Mgode [42], we use in Fig. 1 the name *Acomys* aff. *percivali.*

*Type locality:* Not relevant.

*Karyotype:* 2n = 58 [42].

*Additional information:* Mgode [42] named 13 spiny mice from northern Tanzania (localities Tingatinga, Longido, Mt. Gelai-Olikisima and Kilimamoja-Karatu) belonging to this lineage as *Acomys* cf. *percivali*. They differ from *Wil4* (= *A. wilsoni*) in skull morphology and karyotype. Because the type locality of *A. percivali* (Mt. Nyiro, Kenya) is very far from the distribution of *Wil3*, the name *A. percivali* more probably belongs to *Wil1*, while *Wil3* likely deserves a formal description as a new species.

*(10) Wil4*

*Distribution:* Southern Ethiopia (Somali region), Kenya (east of GRV), north-eastern Tanzania.

*Available name:* *A. wilsoni* Thomas, 1892.

*Type locality:* Mombasa, Kenya.

*Karyotype:* 2n = 62 and NF = 76 [42, 106].

*Additional information:* Verheyen et al. [45] suggested that *A. wilsoni* (meaning *Wil2* and *Wil4* included in their study of mitochondrial variation) is probably a species complex, which is confirmed by our data. The conspecificity of *Wil4* with *Wil2* and/or *Wil3* should be further investigated as they might represent intraspecific phylogeographic structure (see similar patterns in savanna-dwelling rodent species in southern part of Somali region, e.g. in *Gerbilliscus vicinus*; [72] or *Saccostomus umbriventer*; [71]). If *Wil2* and *Wil4* are different species, the analysis of the type material of *A. wilsoni* will be required to decide, which of them is true *A. wilsoni* (both are distributed around the type locality of *wilsoni*).

#### The cahirinus group

This is the most diversified *Acomys* group comprising of three main clades, “Djibuti” (one MOTU *Dji*), “*ignitus*” (four MOTUs *Ign1* − *Ign4*) and “*cahirinus*-*dimidiatus*” (11 MOTUs *Cah1* − *Cah11*), with unresolved mutual relationships.

*(11) Dji*

*Distribution:* Djibuti, Afar province in Ethiopia, probably also Somalia (from where no genetic data are available).

*Available name:* *Acomys louisae* Thomas, 1896.

*Type locality:* 65 km S of Berbera, Somalia.

*Karyotype:* 2n = 68 and NF = 68 ([107]; our unpubl. data).

*Additional information:* *Acomys louisae* was placed in a separate subgenus *Peracomys* based on dental characters [51]. We have not checked the skull morphology of our material from *Dji*, but the recently collected animals from eastern Ethiopia (Dire Dawa region) likely assigned to *A. lousiae* by morphological characters (by C. Denys, unpubl. data) clustered with *Dji* at *CYTB* (not included in this study). According to Petter [108], *A. louisae* cannot be distinguished from the “*cahirinus*-*dimidiatus* complex” (sensu [109, 110]) on the basis of skull or external characteristics. This species may co-occur with *A. mullah* (*= Cah6*) in the Afar triangle (larger, HB > 100 mm with grey or greyish-brown dorsal pelage). *A. louisae* should have a bright rufous or brown dorsal pelage and relatively very long tail (> 100% of HB; [36]). In our pilot analysis we were not able to find significant external size differences between individuals from lineages *Dji* and *Cah6* (considered as *A. mullah*, see below), but more detailed morphological investigation is needed.

*(12) Ign1*

*Distribution:* Eastern part of Ethiopia (Babile).

*Available name:* None. Based on Lavrenchenko et al. [111], we use the name *Acomys* sp. C in Fig. 1.

*Type locality:* Not relevant.

*Karyotype:* 2n = 44, NF = 68 [111, 112].

*Additional information:* This presumably new species was mentioned for the first time as genetically and cytogenetically very divergent lineage (*Acomys* sp. C) by Lavrenchenko et al. [111] from the Babile Elephant Sanctuary in Eastern Ethiopia. This lineage is only known from the Babile Elephant Sanctuary, where it is very common and abundant species. It can have wider distribution in poorly sampled regions of southeastern Ethiopia and Somalia (see similar pattern in gerbils, [72]). The conspecificity with other lineages of the *ignitus* clade should be further tested. The comparison with the type material of *A. mullah*, described from nearby town Harar, is necessary (see also below).

*(13) Ign2*

*Distribution:* South-eastern Ethiopia.

*Available name:* None.

*Type locality:* Not relevant.

*Karyotype:* Not known.

*Additional information:* This lineage is reported here for the first time. It is known only from two localities in the south-eastern slope of Ethiopian Highlands (Sof Omar caves and Imi; each locality has very distinct mitochondrial haplotypes). It might be more widespread in poorly sampled Somali region of Ethiopia, and in Somalia. Its sister lineage *Ign3* (= *A. ignitus*) is geographically distant, but the conspecificity with other lineages of the *ignitus* clade are worth of further taxonomic work.

*(14) Ign3*

*Distribution:* Southern Kenya, northernmost Tanzania.

*Available name:* *Acomys ignitus* Dollman, 1919. In Alhajeri et al. [46] was this species incorrectly mentioned as *A. percivali*.

*Type locality:* Voi, Kenya.

*Karyotype:* 2n = 50, NF = 66–68 [102].

*Additional information:* *A. ignitus* has been recorded in and around Tsavo National Park [36], which corresponds to the distribution of this MOTU. Whether or not other lineages of the *ignitus* clade (especially *Ign2*) are conspecific with *A. ignitus* must be investigated by integrative taxonomy approach.

*(15) Ign4*

Distribution: Kenya and southernmost Ethiopia (east of GRV).

*Available name:* *Acomys kempi* Dollman, 1911.

*Type locality:* Chanler Falls, N Guaso Nyiro, Kenya.

*Karyotype:* Not known.

*Additional information:* This species was previously listed as subspecies of *A. ignitus* (Hollister, 1919) [113] or *A. cahirinus* (Setzer, 1975) [38], but rehabilitated as clearly distinct species by Janecek [103]. *Acomys kempi* was found sympatric with *A. percivali* (= *Wil1*) at several localities in Kenya and southern Ethiopia and these two taxa can be easily distinguished, e.g. by coat coloration (the latter being usually greyish and darker). There is no evidence of distributional overlap with *Ign3* (*A. ignitus*), but further sampling in southern Kenya would be desirable.

*(16) Cah1*

*Distribution:* North-west Kenya (the only region from where the genetic data is available), Sudan, South Sudan. Very probably also in Uganda.

*Available name:* *Acomys cineraceus* Heuglin, 1877.

*Type locality:* Doka, Sudan.

*Karyotype:* 2n = 48–50 [114].

*Additional information:* Formerly included in *A. cahirinus* [38, 108], but Dieterlen (in litt.) noted that *A. cineraceus* is a distinct species [37]. Separation of *A. cineraceus* from *A. cahirinus* is supported by chromosomal data (2n = 48 or 50 for *A. cineraceus*, 2n = 36 for *A. cahirinus*; [37, 114]). Limits of the geographic range of *A. cineraceus* are unresolved, especially its western part [37] . Compared to the distribution maps in Denys et al. [16] and Happold [115], we were not able to confirm its occurrence in western Ethiopia.

*(17) Cah2*

*Distribution:* Burkina Faso, Mali.

*Available name:* None. Based on Barome et al. [30], we call this MOTU as *Acomys* sp. 2 in Fig. 1.

*Type locality:* Not relevant.

Karyotype: 2n = 66–68, NF = 66–72 [116].

*Additional information:* Barome et al. [30] called this MOTU as *Acomys* sp. 2, Granjon and Duplantier [116] included it in *A. johannis* species complex. The distribution of *Cah2* in West Africa is overlapping with *Cah3* and *Cah4*, and its specific status should be investigated by integrative taxonomy approach using larger material and multi-locus genetic analysis.

*(18) Cah3*

*Distribution:* Burkina Faso.

*Available name:* None. Based on Barome et al. [30], we call this MOTU as *Acomys* sp. 1 in Fig. 1.

*Type locality:* Not relevant.

*Karyotype:* 2n = 66–68, NF = 66–72 [116].

*Additional information:* Barome et al. [30] called this MOTU as *Acomys* sp. 1, Granjon and Duplantier [116] included it in *A. johannis* species complex. The distribution of *Cah3* in West Africa is overlapping with *Cah2*, *Cah4* and *Cah11*. The West African *A. johannis* species complex (paraphyletic in our study, grouping *Cah2*, *Cah3* and *Cah4*; see also [116]) requires taxonomic revision.

*(19) Cah4*

*Distribution:* Chad, Cameroon, Niger, Nigeria, Benin.

*Available name:* *A. johannis* Thomas, 1912.

*Type locality:* Bauchi Plateau Kabwir, North Nigeria.

*Karyotype:* 2n = 66–68, NF = 66–72 [116].

*Additional information:* This MOTU was included in previous phylogenetic studies as *A. johannis* [30, 31, 37, 43, 46]. Formerly it was included in *A. cahirinus* [38] or *A. cineraceus* [104]. Sicard and Tranier [63] provided a detailed report on the geographic distribution of three pelage colour phenotypes of *Acomys* occurring in Burkina Faso [117], assigned them to *A. johannis*, and contrasted their external, cranial, and dental morphology with *A. chudeaui* (= *Cah11*). Using *CYTB* sequences, Barome et al. [31] reported the specimens from Burkina Faso and Mali as *Acomys* sp. 1 (= *Cah3*) and *Acomys* sp. 2 (= *Cah2*) and specimens from Niger, Benin, Cameroon, and Niger as *A. johannis* (= *Cah4*), but their conspecificity has never been tested by the combination of multi-locus genetic and phenotypic data.

*(20) Cah5*

*Distribution:* Egypt (Sinai only), Arabian Peninsula, South Iran.

*Available name:* *Acomys dimidiatus* Cretzschmar, 1826.

*Type locality:* Sinai, Egypt.

*Karyotype:* 2n = 36–38, NF = 68–70 [110].

*Additional information:* *Acomys dimidiatus* is nearly indistinguishable from *A. cahirinus* with regard to external morphology, which resulted in great confusions relative to its classification [16]. With few exceptions (e.g.*,* [34, 37, 38, 46, 118, 119]) *A. dimidiatus* usually has been listed in the synonymy of *A. cahirinus* [36, 37, 82, 120]. A morphological and cytogenetical review of *Acomys* species made by Denys et al. [51], who provided the external, skull and dental characteristics of all the type specimens available, validated *A. cahirinus* as distinct from *A. dimidiatus*. Frynta et al. [33] referred two major lineages in northern Africa and Middle East, which should represent *A. cahirinus* and *A. dimidiatus*, respectively. The type localities of these two species are very close each other (Cairo and Sinai) but their distributions seem to be separated by the Isthmus of Suez.

*(21) Cah6*

*Distribution:* Horn of Africa (S Eritrea, Djibouti, E Ethiopia and N Somalia).

*Available name:* *Acomys mullah* Thomas, 1904.

*Type locality:* Harar, Ethiopia.

Karyotype: Not known.

*Additional information:* Awaiting more detailed taxonomic revision, we assigned the name *A. mullah* to the lineage *Cah6* distributed in the margins of the Afar triangle in Ethiopia. This species is considered a member of the “*cahirinus*-*dimidiatus*” complex [16, 121], which is confirmed by our phylogenetic study. This species may co-occur with *A. louisae* (= *Dji*). It should be also taken in consideration that the type locality of *A. mullah* (Harar) is very close to the only known locality of *Ign1* and is separated from the Afar lowland by the Chercher Mts. Comparison of *Ign1* and *Cah6* with the type material of *A. mullah* (and *A. brockmani* considered as its synonym) is necessary.

*(22) Cah7*

*Distribution:* North-west Ethiopia (Mai Temen and Alatish NP).

*Available name:* None. Based on Lavrenchenko et al. [112], we use the name *Acomys* sp. B in Fig. 1.

*Type locality:* Not relevant.

*Karyotype:* 2n = 40, NF = 68 [112].

*Additional information:* Ivlev et al. [25] and Lavrenchenko et al. [112] called this lineage *Acomys* sp. B. It is sympatric with *Cah8*, but the two lineages significantly differ by karyotypes, physiological and behavioural traits [112] and thus might represent different biological species.

*(23) Cah8*

*Distribution:* Alatish NP, Ethiopia.

*Available name:* None. Based on Lavrenchenko et al. [112], we use the name *Acomys* sp. A in Fig. 1.

Type locality: Not relevant.

*Karyotype:* 2n = 52, NF = 68 [112].

*Additional information:* Ivlev et al. [25] and Lavrenchenko et al. [112] called this lineage *Acomys* sp. A. It is also very abundant in the neighbouring Dinder NP in Sudan (J. Bryja et al., unpublished data). The taxonomic revision of *Cah7*, *Cah8*, *Cah9* and *Cah10* is necessary and more intensive sampling in Sudan and northern Ethiopia would be very helpful.

*(24) Cah9*

*Distribution:* Egypt, Greece (Crete), Cyprus, Turkey, Libya, northern Chad.

*Available name:* *Acomys cahirinus* (É. Geoffroy Saint-Hilaire, 1803).

*Type locality:* Cairo, Egypt.

*Karyotype:* 2n = 36–42, NF = 68 [54, 110].

*Additional information:* The species was described from Cairo (Egypt). It seems very likely that it colonized eastern Mediterranean area during Antiquity. Weak genetic differences revealed also by our multilocus analysis support the view that *A. cahirinus* should be synonymized with *A. minous* Bate, 1906 from Crete, *A. cilicicus* Spitzenberg, 1978 from Turkey and *A. nesiotes* Bate, 1903 from Cyprus (see [16, 54] and references there). The relationships with *A. seurati* (a distinct taxon from rocky areas in southern Algeria, differing by karyotype and dental morphology; [51]) should be investigated by using genetic data from the Algerian material.

*(25) Cah10*

*Distribution:* Sheraro, Ethiopia.

*Available name:* None.

*Type locality:* Not relevant.

*Karyotype:* Not known.

*Additional information:* *Cah10* is known only from one locality in North Ethiopia. It is a sister MOTU either to *Cah9* or *Cah11* and its conspecificity with *A. cahirinus* and/or *A. chudeaui* should be tested.

*(26) Cah11*

*Distribution:* Niger, Mauritania, Mali, Chad.

*Available name:* *Acomys chudeaui* Kollman, 1911.

*Type locality:* Atar, SW of Biskra, Mauritania.

*Karyotype:* 2n = 40–46, NFa = 66 [116].

*Additional information:* This taxon has been previously listed as a synonym of *A. cahirinus*, but most recent works consider it as a distinct species [36, 46, 116]. Nicolas et al. [43] synonymized *A. airensis* and *A. chudeaui* and provided a detailed phylogeographic analysis of this taxon.

## Conclusions

Using multilocus genetic data, comprehensive geographic sampling and multiple phylogenetic approaches, we revealed that the spiny mice (*Acomys*) are composed of five main species groups: *subspinosus, spinosissimus, russatus, wilsoni* and *cahirinus.* Three of them (*spinosissimus*, *wilsoni* and *cahirinus*) clearly represent species complexes. We delimited 26 genetic lineages as potential *Acomys* species, and their taxonomic status should now be assessed by multidisciplinary investigations. The origin of the genus is dated to the late Miocene in savannas of eastern Africa, when the first vicariance between “southern” and “northern” groups was probably caused by the development of the coast-to-coast forest belt. The evolutionary history of the genus in Plio-Pleistocene was influenced by global climatic transitions as well as by local geomorphological features (e.g. deserts, mountain blocks and/or large water bodies) and is characterized by repeated cycles of diversifications, especially in eastern Africa, and repeated dispersal events mainly to the North and West. The spiny mice can be thus used as very suitable model for testing specific hypotheses of the role of historical factors on the formation of current biodiversity of seasonally dry environments of Afro-Arabia.

## Methods

### Sampling

The genetic dataset is based on 700 individuals of spiny mice. We produced original genetic data from 421 individuals collected at more than one hundred localities, and complemented them by 279 georeferenced mitochondrial sequences from GenBank. This material covers large part of the distribution of the genus as predicted by the IUCN [122] (see Fig. 2a). All individuals were DNA-barcoded at mitochondrial markers to get as precise distributional maps of genetic clades as possible, but part of sequences was removed as redundant from subsequent phylogenetic analyses (see Additional file 5). All fieldwork performed in the frame of this study complied with legal regulations in particular countries and sampling was in accordance with local legislation (see more details in Ethics approval section). Rodents were trapped in Sherman live traps (H.B. Sherman Traps Inc., Tallahassee, USA) and snap traps baited with a mixture of peanut butter, maize flour and dried fish. Mice caught in live traps were euthanized by cervical dislocation or an overdose of Isoflurane prior to dissection (Directive 2010/63/EU). When present, the spiny mice are generally the most abundant component of the small mammal communities and are not listed as endangered. Each individual was identified to the genus by the external features and the tissue sample (tail, toe, spleen, etc.) was stored in 96% ethanol until DNA extraction. GPS coordinates of each locality were recorded. For more details on particular specimens, localities and collectors, see Additional file 5.

### DNA extraction, amplification and sequencing

DNA from 96% ethanol-preserved tissue samples was extracted using a DNeasy Blood & Tissue kit (Qiagen, Hilden, Germany) following the manufacturer’s instructions. For phylogenetic analysis we selected four genetic markers; two mitochondrial fragments, cytochrome *b* (*CYTB*) and control region (*D-loop*) and two nuclear exons, Interphotoreceptor Binding Protein gen (*IRBP*) and Recombination activating gene 1 (*RAG1*). Individual markers were amplified by the polymerase chain reaction (PCR) using following combination of primers: L14723 and H15915 [123] for *CYTB*; ‘primers 1–4’ [124] for *D-loop*; IRBP217 and IRBP1531 [125] for *IRBP* and RAG1F1705 and RAG1R2951 [126] for *RAG1*. Each locus was amplified using a final concentration of 3 mM of MgCl_2_ (for *IRBP* only 2 mM), 0.2 mM of each dNTP, 0.4 μM of each primer, 1 x Taq buffer (Thermo Fisher Scientific, Waltham, USA), 0.2 μl of Taq polymerase (5 U/μl, Thermo Fisher Scientific), 30 ng/μl of genomic DNA, and ddH_2_O to a total volume of 15 μl. PCR products were purified with Calf Intestine Alkaline Phosphatase and Exonuclease I (New England Biolabs, Ipswich, USA), and Sanger-sequenced in both directions using the BigDye® Terminator chemistry (Thermo Fisher Scientific) either at the Institute of Vertebrate Biology CAS on an ‘Applied Biosystems® 3130xl Genetic Analyzer’ or commercially through the GATC Biotech company (Konstanz, Germany). All corresponding sequences were deposited in GenBank under accession numbers MH044731-MH045045 (see Additional file 5).

### Phylogenetic analysis

The final dataset for phylogenetic analyses consisted of 373 unique sequences of *CYTB*, 71 sequences of *IRBP*, 59 sequences of *RAG1* and 96 sequences of *D-loop.* The remaining mitochondrial sequences (usually identical and/or shorter sequences and/or from the same or neighbouring localities) were unambiguously assigned to particular mtDNA lineages by preliminary phylogenetic analysis and they were removed as redundant (see Additional file 5). These data were used only to increase the precision by which we mapped the geographical distribution of phylogenetic clades. Nuclear exons were sequenced only in the representative subset of 102 individuals, covering the geographic distribution and mitochondrial diversity as much as possible (see Additional files 5 and 6). As outgroups we used four taxa from subfamily Deomyinae, to which *Acomys* belong (*Deomys ferrugineus, Lophuromys flavopunctatus, Lophuromys sikapusi* and *Uranomys ruddi*; see Additional file 5). Sequences were aligned in MUSCLE [127] and the concatenated dataset with total length 4005 bp was created in Mesquite. For all three protein-coding genes (*CYTB*, *IRBP*, *RAG1*), we used Mesquite 3.03 [128] to check the coding frame for possible errors or stop codons.

Phylogenetic reconstructions were conducted using maximum likelihood (ML) and Bayesian inference (BI). For both phylogenetic approaches were carried out partitioned analyses to improve phylogenetic accuracy [129]. The molecular dataset was divided into ten partitions: we used three partitions for each of the protein-coding genes, and one partition for the control region (*D-loop*). The best partitioning scheme and substitution models were determined with PartitionFinder v1 [130] using a greedy heuristic algorithm with ‘linked branch lengths’ option. The Bayesian information criterion (BIC) was used to compare partitioning schemes and substitution models following the recommendation of Ripplinger and Sullivan ([131]; Table 2).

**Table 2:** The substitution models used in particular phylogenetic analyses. They were selected by PartitionFinder using BIC model selection, greedy search, linked branch length

Partitions		RAxML	MrBayes	BEAST
1	*CYTB*_pos1	GTR + I + G	GTR + I + G	GTR + I + G
2	*CYTB*_pos2	GTR + I + G	HKY + I + G	HKY + I + G
3	*CYTB*_pos3	GTR + I + G	GTR + I + G	GTR + I + G
4	*IRBP*_pos1, *RAG1*_pos2	GTR + G	HKY + I	TrN + I
5	*IRBP*_pos2, *RAG1*_pos3	GTR + G	HKY + G	TrN + G
6	*IRBP*_pos3, *RAG1*_pos1	GTR + I + G	HKY + I + G	HKY + I + G
7	*D-loop*	GTR + I + G	GTR + I + G	GTR + I + G

Maximum likelihood analyses were performed using RAxML v8.2.8 [132] for separate gene trees (*CYTB* and *D-loop* not shown same topology as Fig. 1, *IRBP* Additional file 2, *RAG1* Additional file 3) as well as for concatenate matrix. Based on the BIC results in PartitionFinder we used seven partitions for concatenate matrix, five partitions with GTR + I + G model and two with GTR + G substitution model (Table 2). The ML tree was obtained using heuristic searches with 100 random addition replicates and the clade support was then assessed using a non-parametric bootstrap procedure with 1000 replicates. Following Hillis and Bull [133], nodes supported by bootstrap values (BP) ≥ 70 were considered strongly supported.

Bayesian inference analyses were carried out using MrBayes v3.2.6 [134] with seven partitions (Table 2). Two independent runs with four MCMC (one cold and three incrementally heated) were conducted: they ran for 50 million generations, with trees sampled every 1000 generations. A conservative 25% burn-in was applied after checking for stability on the log-likelihood curves and the split-frequencies of the runs. Support of nodes for MrBayes analyses was provided by clade posterior probabilities (PP) as directly estimated from the majority-rule consensus topology. Following Erixon et al. [135], nodes supported by PP ≥ 0.95 were considered strongly supported.

### Estimates of species richness

For estimation of *Acomys* species richness we applied multiple species delimitation methods (as suggested by Carstens et al. [136]): (1) “by-eye” analysis of genetic structure (based primarily on *CYTB* barcodes) and geographical distribution of genetic lineages; (2) species discovery approach to assign individuals to putative groups based on the variability of *CYTB* sequences [137, 138]; (3) species delimitation based on multi-locus data and multispecies coalescent methods [139].

In the first simplest approach, we produced ML tree based only on *CYTB* sequences. We then compared the revealed clades with the species names used in previous studies (e.g. [31, 32, 41, 43, 45]), and the distribution of particular clades with positions of type localities of nominal species. By using this approach we newly identified several highly supported phylogenetic clades with parapatric distribution to previously analysed and named species, which might represent new species and are worth of further taxonomic studies. Genetic distances (within species and to the genetically nearest lineage) from BI tree were additionally also computed using the species delimitation package [140] implemented in Geneious v9.1.8 [141].

Second, we performed two analyses of species discovery based on diversity of *CYTB* marker. The Automatic Barcode Gap Discovery approach (ABGD; [137]) was used to identify barcode gap between intraspecific and interspecific genetic distances. An alternative Poisson Tree process (PTP) approach models intra- and interspecies processes by directly using the number of substitutions [138]. We used a recently improved algorithm based on PTP, the so-called multi-rate PTP (mPTP; [142]), which works better for phylogenies that have different rates of speciation-coalescence and allows to account for the different rates of branching events within each delimited species [142]. Both analyses (ABGD and mPTP) were performed using the ultrametric *CYTB* phylogeny produced by Bayesian method with strict clock in BEAST v2.4.7 [143].

The last used species delimitation approach, Species Tree And Classification Estimation, Yarely (STACEY; [139]), is a Bayesian method based on the multispecies coalescent model and estimates the probability of distinct species delimitation hypotheses given multilocus data. By utilizing multi-species coalescent theory and phylogenetic inference under a full probability Bayesian network, STACEY simultaneously estimates gene trees, the species tree and species delimitations under the assumption that all individuals that are affected by the same coalescent process, also belong to the same species/clade. We assumed conspecificity of individuals bearing mtDNA of the same lineage, identified by the first arbitrary approach described above. All but one lineages were represented at least by two individuals genotyped minimally at 3–4 markers (see Additional file 5). To relax the prior assumptions about species delimitation, we estimated a species tree using the birth-death-collapse model [144] as implemented in STACEY for BEAST 2 [145]. STACEY does not require guide tree, therefore errors resulting from a priori phylogenetic assumptions are avoided. Parameters and priors for the analysis were set according to the recommendations of STACEY manual [139]. Sequence alignments were imported into BEAUTI where they were assigned separate and unlinked substitution, clock and tree models. For mitochondrial markers (*CYTB + D-loop*) the ploidy was set of 0.5, for nuclear genes (*IRBP, RAG1*) 2.0. For the species tree prior the collapse height was set to 10^− 3^. Three independent MCMC chains were run for 10^− 7^ generations and log every 5000 generations. The burn-in was 25%, and the outputs from three runs were combined in LogCombiner 2.4.7 [146]. The similarity matrix was created using SpeciesDelimationAnalyser version 1.8.9 [147] with 25% burn-in and collapse height of 10^− 3^. Species tree was visualised as a cloudogram using DensiTree [148].

### Divergence dating

To calibrate a molecular clock, we compiled the set of usable fossils for the genus *Acomys* and its ancestors: (1) an extinct genus †*Preacomys* with three described species: †*P. griffini* Mein et al., 2004 and †*P. karsticus* Mein et al., 2004 from Harasib, Namibia (9 Ma; [83, 149]) and †*P. kikiae* Geraads, 2001 from Chorora, Ethiopia (8.5 Ma; [150, 151]). Because the position of these three fossils on phylogenetic tree is not unequivocally clear, we used a minimum age 8.5 Ma as a root for the genus *Acomys*. (2) *Acomys* from Lemudong’o locality in Kenya (6.08–6.12 Ma; [152, 153]) is the oldest *Acomys* and we considered it as the most recent common ancestor (MRCA) for taxa living currently in the northern part of eastern Africa (with the centre of their distribution in Somali-Masai savanna) and in Arabia, i.e. the clade encompassing *cahirinus + wilsoni + russatus* groups. (3) The oldest fossil of the *spinosissimus* group (sensu Verheyen et al. [45]) was discovered in Transvaal, South Africa (3 Ma; [154]), and we used it as MRCA for this group. Bayesian analyses of divergence dating were conducted on a species tree in *BEAST v2.4.7 [143]. The species were defined based on STACEY results. The mitochondrial (*CYTB + D-loop*) and nuclear genes (*IRBP, RAG1*) were imported in BEAUTI where they were assigned separate and unlinked substitution, clock and tree models. Bayesian analysis run with uncorrelated lognormal relaxed clocks [155], birth-death tree prior [156] and selected fossil constraints were defined by using lognormal statistical distributions (see Table 3 for more details). Two independent runs were carried out for 10^7^ generations with sampling every 1000 generations in BEAST. We discarded first 25% as burn-in and the resulting parameter and tree files were examined for convergence and effective sample sizes (> 200) in Tracer 1.6 [157]. The two runs were combined in LogCombiner and the species tree was visualized in TreeAnotator.

**Table 3:** List of fossils associated with the genus *Acomys* used in the divergence dating. The offset and mean represent the specification of lognormal priors used for the calibration of molecular clock. All fossil constrains were used as a crown

Fossil	MRCA	Locality	Author	Age (Ma)	Offset	Mean
†*Preacomys kikiae*	*Acomys*	Chorora, Ethiopia	Geraads (2001, 2002); Suwa et al., (2015)	8.5	8.5	1.0
†*Acomys* I. Geoffroy	*cahirinus + wilsoni + russatus*	Lemudong’o, Kenya	Manthi (2007); Manthi and Ambrose (2007)	6.08–6.12	6.08	1.0
†*Acomys spinosissimus* Peters	*spinosissimus*	Transvaal, South Africa	Denys (1999)	3	3	1.0

### Biogeographical reconstructions

The BioGeoBEARS approach [158] was used to reconstruct the ancestral distributions and diversification patterns. Six major biogeographic regions with *Acomys* occurrence were defined on the basis of Holt et al. [159] and Linder et al. [3]: South Africa region (S – South Africa), Zambezian region (Z – Zambezian region), Somali region (E – East Africa), Sudanian region (W – West Africa), Sahara region (N – North Africa) and the Arabian region (A – Arabian region). Dispersal rate between adjacent areas (S-Z, Z-E, E-W, E-N, N-A) was fixed to 1, whereas the dispersal of 0.5 (S-E, Z-W, Z-N, E-A) was defined for long distance dispersal (i.e. biogeographical areas separated by another region) or whenever a geographical barrier had to be crossed (e.g. multiple water bodies). Dispersal was disallowed between geographical areas separated by two or more areas (S-W, S-A, S-N, Z-N, Z-A, W-A). Biogeographic reconstruction relied on the Dispersal-Extinction Cladogenesis model (DEC) of range evolution [160]. DEC model estimates geographical range evolution using a phylogenetic tree with branch lengths scaled to time, geographical (habitat) areas for all tips, and an adjacent matrix of plausibly connected areas [85]. Because of concerns with its statistical validity [161] we did not use the +J model of Matzke [158] in our analyses.

### Species distribution modelling

Assuming phylogenetic niche conservatism [162] and generally similar ecological requirements for all taxa of spiny mice, we modelled the present and past distribution of suitable climatic conditions for the genus *Acomys* by the maximum entropy approach [163]. We used 282 presence records (unique localities, Additional file 5, Fig. 2) as the input data to train the model. We modelled the suitable conditions in the recent, but we also produced paleoclimatic projections for the last glacial maximum (21 ka; MIROC resolution 2.5 min [164]) and for the last interglacial (120–140 ka; resolution 30 s [165]) using 19 bioclimatic variables from the WorldClim database [166]. The background was restricted to whole Africa and Arabia (Figs. 2 and 3). The species distribution modelling (SDM) analysis was performed using MaxEnt v3.3.3 k [167]. We used 10 replicates and the importance of environmental variables was tested using jackknife option, and for the regularization multiplier we used the default value of 1. The SDM results were converted in a map using QGIS with a maximized sum threshold [168, 169].

## Additional files

Additional file 1: Taxonomic classifications of the genus *Acomys*. Bold italic names represent species reported in particular lists, standard italics represent subspecies (in Ellerman [34]; Setzer [38]; Denys et al. [16]). (XLSX 41 kb)

Additional file 2: ML phylogenetic tree based on *IRBP* sequences (nexus file). (NEXUS 6 kb)

Additional file 3: ML phylogenetic tree based on *RAG1* sequences (nexus file). (NEXUS 5 kb)

Additional file 4: Maximum clade credibility tree from STACEY with PP support (nexus file). (NEXUS 24 kb)

Additional file 5: Complete list of individuals used in this study, with details on localities and genetic data. (XLSX 97 kb)

Additional file 6: Alignment of 369 ingroup and 4 outgroup concatenated sequences of *CYTB*, *IRBP, RAG1* and *D-loop*. (NEXUS 1470 kb)

## Abbreviations

2n: Diploid number of chromosomes
a.s.l.: Above sea level
ABGD: Automatic Barcode Gap Discovery
BI: Bayesian Inference
BIC: Bayesian information criterion
bp: Base pairs
BP: Bootstrap value
*CYTB*: Cytochrome b gene
DEC: Dispersal-Extinction Cladogenesis model
*D-loop*: Control region
EAM: Eastern Arc Mountains
EARS: The East African Rift System
GRV: The Great Rift Valley
HPD: Highest posterior density
iNHG: The intensification of Northern Hemisphere glaciation
*IRBP*: Interphotoreceptor Binding Protein gen
ka: Thousand years
LGM: Last Glacial Maximum
LIG: Last Interglacial
Ma: Million of years ago
MaxEnt: Maximum entropy
MCMC: Markov chain Monte Carlo
ML: Maximum likelihood
MOTU(s): Molecular operational taxonomic unit(s)
mPTP: Multi-rate Poisson Tree process
MRCA: The most recent common ancestor
MSC: Messinian salinity crisis
Mt. (s): Mountain(s)
mtDNA: Mitochondrial DNA
NF: Fundamental number of chromosome arms
NFa: Fundamental number of autosomal chromosome arms
NP: National park
PCR: The polymerase chain reaction
PP: Posterior probability
PTP: Poisson Tree process
*RAG1*: Recombination activating gene 1
SDM: Species distribution modelling
SRM: Southern Rift Mountains
STACEY: Species Tree And Classification Estimation, Yarely
